# Generalized Vaccinia 2 Days after Smallpox Revaccination

**DOI:** 10.3201/eid0912.030592

**Published:** 2003-12

**Authors:** James R. Miller, Nick M. Cirino, Edward F. Philbin

**Affiliations:** * New York State Department of Health, Albany, New York, USA; † Albany Medical College, Albany, New York, USA

**To the Editor:** Hospital and public health personnel are currently receiving smallpox vaccination in a national effort to increase preparedness for a possible deliberate release of smallpox (*1*). Generalized vaccinia (GV) is a typically self-limited adverse event following vaccination (incidence 23.4–238.2 cases per million primary vaccinees and 1.2–10.8 cases per million revaccinees) (*2*,*3*).

We report the clinical course and laboratory diagnosis of GV in a 37-year-old woman with a history of at least one uncomplicated childhood inoculation that left a vaccination scar. She was revaccinated on March 12, 2003. Before revaccination, the patient reported no contraindications to vaccination and denied any conditions that typically weaken the immune system (including HIV/AIDS, leukemia, lymphoma, other cancers, radiation, chemotherapy, organ transplant, posttransplant therapy, immunosuppressive medications, severe autoimmune disease, and primary immune deficiency). The patient also confirmed that she did not have a skin disease or a history of eczema or atopic dermatitis, nor was she pregnant or allergic to a vaccine component.

On March 14, some 44 hours after vaccination, the patient reported headache, chills, pruritus, chest pain (described as chest “heaviness”), recurrent vomiting, and maculopapular lesions. The lesions, characterized by the patient as “mosquito bites,” first appeared on the face, then the legs, and then the trunk and upper extremities. Maximum oral temperature was 37.7°C. Over the next 4 days, approximately 30 pustules developed, several of which began to drain. Nausea persisted, and the patient had a stiff neck and recurring chest tightness, but physical examination, echocardiography, electrocardiography, and chest radiography were within normal limits. By March 25, the patient’s lesions had all scabbed, the scabs had fallen off, and she felt well enough to return to work. Pustular material obtained on March 18 from two unroofed lesions on the shoulder (Figure) and back tested positive at the Wadsworth Center-Axelrod Institute, New York State Department of Health, for vaccinia virus DNA by a TaqMan (Applied Biosystems, Foster City, CA) real-time polymerase chain reaction assay provided by the Laboratory Response Network, Centers for Disease Control and Prevention. The presence of orthopoxvirus was confirmed by electron microscopy of lesion fluid.

**Figure:**
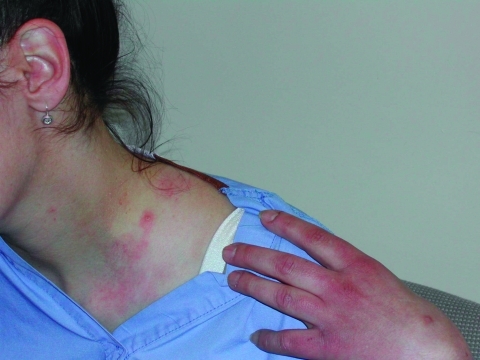
Pustular lesion on patient’s shoulder, 6 days after revaccination.

This case is the first report of a laboratory-confirmed case of GV among recent civilian vaccinees and is notable for the GV occurrence in a revaccinee. GV was not reported among 132,656 military personnel recently revaccinated (*4*). A single case of GV in a revaccinee among 38,514 recent civilian vaccinations (*5*) yields a ratio that exceeds the rate in revaccinees observed in earlier reports and the difference would be even greater if civilians who received primary vaccinations were excluded.

This laboratory confirmation of GV demonstrates the potential of laboratory testing to determine the cause of a post-vaccination rash. Possible cases of GV in earlier surveillance efforts represented a mixed group of rashes, some of uncertain etiology (*6*). This patient’s clinical course is notable for the onset of GV 2 days after vaccination, as compared to a mean of 9 days (range 1–20+) after (generally primary) vaccination (*2*) and suggests that viremia can occur quickly after vaccination.
